# Whole-Genome Sequencing of an Unusual Human Papillomavirus (HPV71) from Latin America (Brazil)

**DOI:** 10.1128/MRA.00343-20

**Published:** 2020-06-11

**Authors:** Edivaldo Costa Sousa Junior, Allan Kaio Silva, Laryssa Danielle da Silva Reis, Lana Patricia da Silva Fonseca, Fabiano Reis da Silva, Fabiolla da Silva dos Santos, Jessylene Almeida Ferreira, Clayton Pereira Silva de Lima, Michelle Carvalho de Abreu, Rodrigo Vellasco Duarte Silvestre, Marcio Roberto Teixeira Nunes

**Affiliations:** a Section of Virology, Evandro Chagas Institute, Ministry of Health, Ananindeua, Brazil; b Center of Technological Innovation, Evandro Chagas Institute, Ministry of Health, Ananindeua, Brazil; DOE Joint Genome Institute

## Abstract

We report the complete genome sequencing of human papillomavirus 71 from Latin America (Brazil).

## ANNOUNCEMENT

The *Papillomaviridae* family is composed of 53 genera, and 5 of them (*Alphapapillomavirus*, *Betapapillomavirus*, *Gammapapillomavirus*, *Mupapillomavirus*, and *Nupapillomavirus*) can infect humans (1). Within the *Alphapapillomavirus* genus, human papillomavirus 71 (HPV71) is associated with mucosal basal keratinocyte infections (2). The genome is a double-stranded circular DNA with a size of 8,017 to 8,038 kb and eight genes enclosed in a nonenveloped icosahedral capsid structure with a diameter of 52 to 55 nm (3). In Brazil, the epidemiology of HPV71 is still unclear, with inconclusive descriptions in the literature even about its relationship to cancer (4–6).

Here, we describe the complete genome sequencing of an infection identified by molecular tools in a Brazilian patient (59 years of age, from the Amazon region) with inflammatory cytology. The genomic DNA was extracted from a clinical sample (cervical smear) that had been collected from a 59-year-old female patient and stored in a solution buffer until DNA extraction using a QIAamp DNA extraction kit (Qiagen, Germany). The genomic library was prepared using the Nextera XT DNA sample preparation kit (Illumina, USA). The quality of the library was verified using a Bioanalyzer 2010 (Agilent Technologies), and the library was sequenced on a HiSeq 2500 instrument (Illumina, USA) with a 2 × 100-bp paired-end format sequencing kit v.4. All laboratory procedures were performed according to the manufacturer’s instructions. The DNA sequencing generated 40,278,934 reads, which were assembled using a *de novo* strategy with MEGAHIT software v.1.2.9 (7), producing 140,912 contigs. These contigs were taxonomically annotated with Kraken v.1 (8), and 10 were related to *Alphapapillomavirus 14* (HPV71), presenting 98.4% nucleotide identity to the HPV71 reference (GenBank accession number NC_039089). The contigs were mapped to the reference sequence (NC_039089) to generate a scaffold. The scaffold generated was about 8,041 kb and was used for reference mapping. We performed reference mapping with SOAP3-dp (9) and generated a consensus sequence using BCFtools (10). The prediction of open reading frames (ORFs) and functional annotation were automatically performed with Geneious v.8.1.9 (11) (similarity, 94%) using a sequence database with 660 annotated papillomavirus genomes retrieved from the Papillomavirus Episteme (PaVE) (12). The genome was manually curated by comparison of the coding ORFs with these genomes using Geneious v.8.1.9 (11). Our final result was the assembly of a genome that is similar to that of the closest species, isolate Qv21030 (AY330620), with 99.84% nucleotide identity, determined using Geneious v.8.1.9 (11). All tools were used with default parameters unless otherwise specified. The genome showed a circular double-stranded structure about 8,033 bp long, with eight fully identified genes and a GC content of 44.4%. The genome identified had a mean coverage of about 25.1× (ranging from 6× to 191×), with the reads overlapping at both ends, forming a circular contig containing the complete genome sequence.

The genome obtained will allow us to contribute to genomic studies with other HPV71 isolates already described throughout the world and will facilitate better understanding of the pathogenic and epidemiological aspects of HPVs in Latin America.

### Data availability.

The complete genome sequence for Brazilian HPV71 has been deposited in GenBank under the accession number MT250602 and in the SRA under accession number SRR11440049.
